# Some Medical Benefit Changes

**Published:** 1924-02

**Authors:** 


### SOME MEDICAL BENEFIT CHANGES.

THE changes in the Regulations governing the arrangements for Medical Benefit which have just come into operation, though not of the same magnitude or importance as those made in 1920, are considerable, and arise from a general revision of the conditions of the insurance medical service. As in 1920, the general scheme and the framework of the panel service remain unaltered; the changes made are changes of detail. In general, they involve a tightening-up of the provisions directed against the erring doctor, they may be said to have the good will of the doctors' representatives, and in some cases have been introduced at their request.

### Free Choice of Doctor.

The insured person may now change his doctor at any time by simply taking his medical card to another practitioner and getting him to sign it. In the past he could only change at the end of a half-year, unless both doctors gave their consent. The medical profession themselves asked for this change—wisely, as we believe. Experience in Manchester has shewn that the number of persons who are likely to change their doctor capriciously is very small. In private practice, people seem usually reluctant to change their doctor. It is, at any rate, satisfactory that, in regard to change of doctor, the conditions have been brought into line with those of private practice. The doctor will have a corresponding right to get rid of a troublesome patient, subject to safeguards against his doing so improperly in the middle of an illness.

### Limitation of Lists.

The maximum number of insured persons for whom a practitioner single-handed may accept responsibility is now 2,500 instead of 3,000, but the Committee have a discretion in exceptional individual cases to raise the limit to 3,000, or to fix a limit lower than 2,500. Special provisions deal with the cases of partnerships and assistants. The Committee may also impose a lower limit than that in force in the area generally, for an individual doctor found to be in default. This provision is a valuable one, but the general limitation of lists affects a few doctors only, interferes with the principle of free choice of doctor, and operates very unequally because of the unknown extent of a doctor's private practice, and of the differences in individual capacity. If, however, there must be a general limit, 2,500 seems sufficiently high.

### Investigation of Complaints.

It is now provided definitely that a doctor shall not carry on his insurance practice elsewhere than at his residence unless he satisfies the Insurance Committee that the arrangements made by him will enable him to discharge his obligations adequately. A certain elasticity is thus provided for, but in urban areas Committees may be expected to insist generally upon a resident caretaker and telephonic communication, proper notices to the patients and adequate deputising arrangements. Without these, the lock-up surgery will always be a reproach to the panel service. In future the Panel Committee may require the Insurance Committee to have an investigation made, either into the conduct of an individual doctor or into any matter affecting the administration of Medical Benefit in the area. This provision, supplementing the existing provisions for complaints by doctors and by insured persons, may place the Panel Committee in an invidious position, but we hope that they will show no lack of moral courage in the matter and prove that they are in earnest about purging the panel service of undesirable elements. The new Regulations also contain a provision that separate complaints established against an individual doctor may have a cumulative effect when the question of penalty arises for determination.

### Practices Lapsing.

It has sometimes happened that a difficulty has arisen as to the most effective way of dealing with the patients of a doctor whose practice has begun to drift through his continued absence or through some bodily or mental disability. In future, where the Committee are satisfied that this is the case, they may simply notify the insured persons on the doctor's list that he is no longer in a position to treat them.

### The Range of Service.

A good deal of rather loose criticism has been directed in the past against the definition of the service to be given, viz., one within the competence of a general practitioner of ordinary competence and skill, and we now find a new definition of what seems to be the same thing. The service will continue to be a " general practitioner " service. The treatment to be given is to comprise " all proper and necessary medical services other than those involving the application of special skill and experience of a degree or kind which general practitioners as a class cannot reasonably be expected to possess." A panel doctor rendering a specialist service, for which he charges a fee, must give evidence of the possession of special skill and experience. Even in such a case if he needs the services of an anæsthetist he must provide it free of charge.

### Other Changes.

Other changes, tending to more efficient administration and the smoother working of the machinery, relate to the cost of prescribing and the giving of certificates of incapacity. As to the former, the Panel Committee will remain arbiters on the question whether there has been extravagance in prescribing, but doctors will first be given the opportunity of threshing the matter out with an Officer of the Ministry in their own homes, and thus possibly save the bother and expense of the more formal investigation by the Panel Committee. In certification, the provisions include a new form of voluntary certificate, a form for a certificate of death, and a requirement that the doctor's name and address should be clearly stamped on all forms. There is a new provision affecting fee-charging, but this subject generally merits fuller treatment and will be reserved for a separate article.

